# Development and validation of a machine learning-based readmission risk prediction model for non-ST elevation myocardial infarction patients after percutaneous coronary intervention

**DOI:** 10.1038/s41598-024-64048-x

**Published:** 2024-06-11

**Authors:** Yanxu Liu, Linqin Du, Lan Li, Lijuan Xiong, Hao Luo, Eugene Kwaku, Xue Mei, Cong wen, Yang Yang Cui, Yang Zhou, Lang Zeng, Shikang Li, Kun Wang, Jiankang Zheng, Zonglian Liu, Houxiang Hu, Rongchuan Yue

**Affiliations:** 1 Department of Cardiology, Affiliated Hospital of North Sichuan Medical College, No. 63, Wenhua Road, Nanchong, 637000 Sichuan Province People’s Republic of China; 2 Department of Cardiology, People’s Hospital of Guang’an District, Guang’an, 638550 People’s Republic of China; 3 School of Pharmacy, Institute of Material Medica, North Sichuan Medical College, Nanchong, 637000 Sichuan People’s Republic of China; 4 Family Health University College and Hospital, Opposite Kofi Annan International Peace Keeping Training Center, Teshie, Accra, Ghana

**Keywords:** Non-ST elevation myocardial infarction, PCI, Readmissions, Prediction model, Machine learning, Cardiology, Risk factors, Signs and symptoms, Diseases, Cardiovascular diseases, Health care, Disease prevention, Health policy

## Abstract

To investigate the factors that influence readmissions in patients with acute non-ST elevation myocardial infarction (NSTEMI) after percutaneous coronary intervention (PCI) by using multiple machine learning (ML) methods to establish a predictive model. In this study, 1576 NSTEMI patients who were hospitalized at the Affiliated Hospital of North Sichuan Medical College were selected as the research subjects. They were divided into two groups: the readmitted group and the non-readmitted group. The division was based on whether the patients experienced complications or another incident of myocardial infarction within one year after undergoing PCI. Common variables selected by univariate and multivariate logistic regression, LASSO regression, and random forest were used as independent influencing factors for NSTEMI patients’ readmissions after PCI. Six different ML models were constructed using these common variables. The area under the ROC curve, accuracy, sensitivity, and specificity were used to evaluate the performance of the six ML models. Finally, the optimal model was selected, and a nomogram was created to visually represent its clinical effectiveness. Three different methods were used to select seven representative common variables. These variables were then utilized to construct six different ML models, which were subsequently compared. The findings indicated that the LR model exhibited the most optimal performance in terms of AUC, accuracy, sensitivity, and specificity. The outcome, admission mode (walking and non-walking), communication ability, CRP, TC, HDL, and LDL were identified as independent predicators of readmissions in NSTEMI patients after PCI. The prediction model constructed by the LR algorithm was the best. The established column graph model established proved to be effective in identifying high-risk groups with high accuracy and differentiation. It holds a specific predictive value for the occurrence of readmissions after direct PCI in NSTEMI patients.

## Introduction

Percutaneous coronary intervention (PCI) has become the most important method for recanalization of myocardial infarction (MI). Advances in revascularization strategies and determination of myocardial reperfusion time window have significantly improved the survival rate of patients with acute myocardial infarction^1^. For this reason, in China and even around the world, the number of PCI operations is increasing year by year. In 2020, the number of patients undergoing coronary intervention therapy in China will reach 1,014,266^2^. However, for patients with MI, the improvement of prognosis is different between STEMI and NSTEMI patients. Compared with STEMI, NSTEMI patients have higher readmissions and mortality rates after discharge^3^.

NSTEMI patients are usually re-admitted due to re-MI and MI complications after MI, resulting in low quality of life and increased economic burden for these patients^4^. In clinical practice, it is difficult for medical staff to predict the prognosis of NSTEMI patients after PCI due to the large changes in the condition of NSTEMI patients and the impact of individual differences such as age, physique, and underlying diseases. Therefore, it is of great significance to effectively identify high-risk groups and reduce the readmission rate of such patients as much as possible. At present, there are few relevant prediction models for the readmission risk of NSTEMI patients after PCI, and it is of great significance to establish this prediction model to accurately identify such high-risk groups to reduce the readmission rate, as well as the medical, social, and economic burden of patients.

Current research on prediction models show that the statistical methods are mostly based on logistic regression and Cox proportional risk regression models^5–7^. In recent years, ML has gradually gained attention from clinicians. ML is a branch of artificial intelligence that has been developed based on computer technology and big data. With the emergence of medical big data, ML methods have significantly enhanced the performance of predictive models. In previous studies, ML has shown better predictive value than traditional prediction models^8–12^ and has the ability to better sort out and analyze massive patient data. Therefore, ML method is an important tool for current medical research^13–16^. Limitations of regression risk scoring systems, such as parameter assumptions, primary reliance on linearity, and limited capability in examining higher-order interactions^17^, can be overcome by ML methods. And species diversity, such as random forests (RF), decision tree and support-vector machine (SVM), extreme gradient boosting (XGBoost), multilayer perceptron (MLP), etc^13,18–20^. Because of the advantages of ML methods, various ML techniques methods can be applied to establish and compare models to determine the best strategy for predicting the readmission of NSTEMI patients after PCI. This will, provide a foundation for accurately assessing the risk of readmission and the long-term prognosis for these patients.

## Methods

### Data description

The data used in this study were obtained from multiple data sources, including the hospital information system, laboratory information management system, Picture Archiving and Communication System (PACS), and electronic medical records of the Affiliated Hospital of North Sichuan Medical University. A total of 1576 patients diagnosed with NSTEMI and treated with PCI were extracted from the Affiliated Hospital of North Sichuan Medical College between June 2014 and October 2022. NSTEMI is defined according to the European Society of Cardiology (ESC) criteria.

### Study design and patient selection

**Figure 1:**
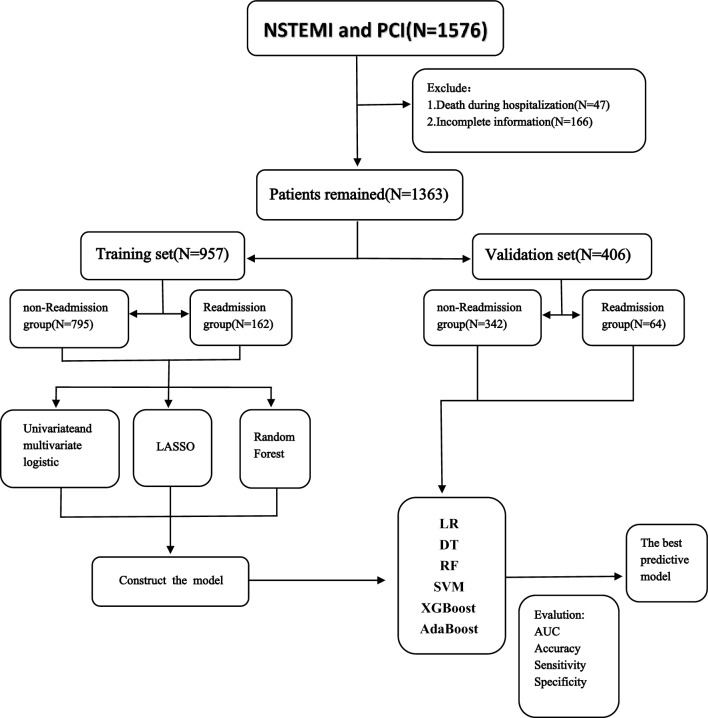
Study design.NSTEMI: Non-ST Elevation Myocardial Infarction; PCI: Percutaneous Coronary Intervention; LASSO: least absolute shrinkage and selection operator; LR: logistic regression; DT: Decision Tree; RF: Random Forest; SVM: support vector machine; XGBoost: extreme gradient boosting; AdaBoost: Adaptive Boosting; AUC: area under the curve.

Between June 2014 and October 2022, a total of 1576 patients diagnosed with NSTEMI were admitted to the Affiliated Hospital of North Sichuan Medical College and underwent PCI treatment. 213 patients were excluded (47 died during hospitalization, and 166 had incomplete data). After exclusion, a total of 1363 patients were included in the study, they were then divided into a training set and a validation set based on a 7:3 ratio, determined by their admission time. In the training set and validation set, the patients were divided into two groups based on whether they were re-hospitalized for myocardial infarction or myocardial infarction-related complications such as heart failure, arrhythmia, etc. (Fig. 1).

### Endpoint and follow-up

The endpoint of this study was readmissions for myocardial infarction or complications related to myocardial infarction (such as heart failure, arrhythmia, etc.) after discharge. Admissions for other diseases not caused by myocardial infarction, such as congenital heart disease, lung disease, and blood system disorders, were not included. The follow-up period was 12 months and was conducted by professional physicians in cardiovascular medicine. The main methods of follow-up were telephone follow-up and medical record follow-up.

### Variable selection

A total of 96 features were considered in this study, which included basic patient characteristics information such as age, gender, BMI, ethnicity, cultural level, etc. Admission clinical features: respiratory rate, systolic blood pressure, diastolic blood pressure, etc. Anamnesis: diabetes, hypertension, stroke, etc. Admission examination and test indicators, such as coronary angiography results, hemoglobin levels, red blood cell counts, platelet counts, D dimer levels, etc. Clinical drugs: such as antiplatelet drugs, antilipids and other drugs. The baseline characteristics of eligible participants are shown in Supplementary Materials (Table S1). To capture a wider range of variables associated with re-admission outcomes within a twelve-month period and to ensure that the variables included in the prediction model were both representative and generalizable, we used three different methods for variable screening in the training cohort. (1) Univariate and multifactorial logistic regression: An inter-group analysis was conducted between the readmitted group and the non-readmitted group. Variables with statistically significant differences between the groups (*P* < 0.05) were included in the multi-factor logistics regression analysis, and then variables with statistical significance (*P* < 0.05) were selected as characteristic variables. (2) The least absolute shrinkage and selection operator regression (LASSO regression) for high-dimensional variable screening and feature selection. LASSO optimizes the coefficients of the regression by adding a penalty term to the standard multiple regression^21^. (3) Random forest method for screening the importance of the outcome event for variables. Random forest is an ensemble learning method that uses bootstrap resampling to construct different trees and combines all the results to form a decision^22^. In this study, the top 20 variables were selected as predictors according to their importance ranking. Finally, the common variables selected by the three methods are taken as the characteristic variables of the model.

### Statistical analysis

SPSS27.0 and R4.2.3 software were used for data processing in this study. We performed descriptive analysis on all patients included in the study. Continuous variables with normal distribution were represented by mean ± standard deviation(SD) and the *t* test was used, continuous variables that did not conform to normal distribution were represented by median (interquartile range) (M (P25, P75)), and comparison between groups was performed by Mann–Whitney U test. The categorical variables were expressed as n (%) and analyzed by Chi-square test, Fisher exact probability method was applied to compare classified data that did not meet the Chi-square test condition. The data set was divided into a training set and a verification set based on the admission time, with an equal sample size ratio of 7:3. The training set was used for variable selection and model construction, while the test set was used for validation. Next, the R4.2.3 software was used to perform logistic regression (LR), Decision Tree (DT), Random Forest (RF), support vector machine (SVM), extreme gradient boosting (XGBoost) and Adaptive Boosting (AdaBoost) six methods to develop the model. Then, the accuracy, sensitivity, specificity, and the area under the curve (AUC), which is the area under the receiver operating characteristic curve (ROC), were estimated and compared to evaluate the performance of the models. This allowed us to find the best model for predicting the re-admission of NSTEMI patients after PCI.

Finally, a nomogram was established according to the optimal prediction model, The calibration curve was used to verify the calibration ability of the model. In addition, in order to verify the clinical practicability of the model, decision curve analysis (DCA) was used to evaluate its clinical benefit. In the graph, the horizontal coordinate is Threshold Probability, which represents the probability of the model predicting re-admission events in the sample. Then readmission measures may do more good than harm, or they may do more harm than good, and the ordinate is the net benefit minus the harm, in addition, there are two special lines, one horizontal solid black line, indicating that all patients have not been readmitted to the hospital, and the net benefit is zero regardless of the probability threshold. A solid gray line showing the change in net benefit as the probability threshold changes when all patients are readmitted. In addition, we further drew the clinical impact curve to visualize its clinical practicability, where the horizontal coordinate is the probability threshold and the vertical coordinate is the number of people, the red line represents the number of people who are judged as high risk by the model under different probability thresholds, and the blue line represents the number of people who are judged as high risk by the model under different probability thresholds and actually have an outcome event, and a loss is added at the bottom: The benefit ratio represents the ratio of loss to gain under different probability thresholds.

All statistical analyses were carried out using SPSS software (version 27), the R programming language (version 4.2.3 (2023–03-15)) and the RStudio software (RStudio 2023.03.0 + 386), involving the “glmnet”,“rpart”,“caTools”,“randomForest”,“xgboost”,“pROC”and “e1071”,“adabag”,“devtools”, “DecisionCurve”, “rmda”packages. The level of statistical significance was *P* < 0.05 (two-tailed).

### Comparison with other models

In order to re-evaluate the accuracy of this model in predicting the readmission of NSTEMI patients after PCI, it was compared with the current commonly used clinical risk scoring models. Three risk scoring models were mainly selected: the adjusted global registry of acute coronary events risk score (adjusted GRACE) model, the Korea Acute Myocardial Infarction Registry risk score (KAMIR) and the Age, creatinine, and ejection fraction score (ACEF). The area under the ROC (AUC) curve was compared using the non-parametric test developed by DeLong et al. MedCalc for Windows version 22.023 (MedCalc Software) was used for comparison.

### Ethical approval

The data used in this study were obtained from multiple data sources, including the hospital information system, laboratory information management system, Picture Archiving and Communication System (PACS), and electronic medical records of the Affiliated Hospital of North Sichuan Medical University. This study was conducted according to the ethical guidelines of the Declaration of Helsinki and approved by the Medical Ethics Committee of the Affiliated Hospital of North Sichuan Medical college (Ethics Approval number: 2023ER322-1). Since the study only involved a retrospective analysis of previous clinical data, the requirement for informed consent was waived by the Ethics Committee of the Affiliated Hospital of North Sichuan Medical College.

## Results

### Baseline characteristics

The study included 1363 study subjects and 96 variables covering basic personal information, vital signs at admission, past medical history, auxiliary examinations and clinical medication. Out of the 1363 patients included in the study, 226 were readmitted within 12 months for a myocardial infarction or complications related to myocardial infarction, on the other hand, 1137 were not readmitted within 12 months. Consequently, the 12-month readmitted rate for MI or MI related complications was 16.58% (supplemental Table S1). In the training cohort, there were a total of 957 individuals, out of which 162 individuals faced re-admission within a 12-month period. This resulted in a re-admission rate of 16.93% (supplemental Table S2). On the other hand, the validation cohort consisted of 406 individuals, with 64 individuals experiencing re-admission within 12 months, resulting in a re-admission rate of 15.76%. (Fig. 1, supplemental Table S3).

The percentage of men and women in the study population varied considerably, with a significantly larger proportion of men than women, probably because of higher smoking rates in men, which in turn led to more severe cardiovascular disease and higher readmission rates. Most of them is the Han nationality. The study participants had an average age of approximately 65 years, almost all of them were admitted through outpatient clinics, and nearly all of them were insured.

### Variable selection

**Figure 2:**
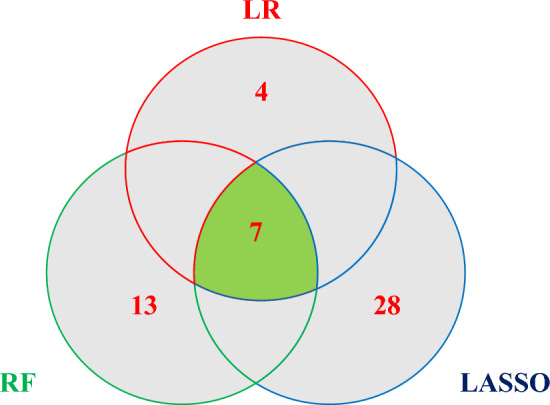
Venn plots reflect the number of results for the three variable screening methods; The overlapping part is the seven variables selected, Logistic regression (LR) non-overlapping part has 4 variables, Random Forest (RF) non-overlapping section has 13 variables, Least absolute shrinkage and selection operator (LASSO) has 28 variables in the nonoverlapping section.

The results of the screening by the three methods are shown below (Fig. 2, Figures S1–S2, Table 1). The statistical parameters of the three variable selection methods are shown in supplemental Table S4. Combining the results of the three methods, we screened seven variables as follows (Table 2). The above 7 variables were all valuable in the three variable screening methods, indicating that they were well represented, so they were used as the characteristic variables to construct the prediction model.

**Table 1:** Univariate and multivariate logistic regression analysis of patients with NSTEMI readmission after PCI surgery.

Readmission of patients with NSTEMI after PCI surgery (training set)				
Variable	Non-readmission (N = 795)	Readmission (N = 162)	Univariate	Multivariate
Age	64.05 ± 11.39	68.74 ± 10.55	< 0.001	< 0.001
Discharge outcomes (ease)	755 (95.0)	124 (76.5)	< 0.001	< 0.001
Mode (walking)	703 (88.4)	106 (65.4)	< 0.001	< 0.001
Communication (good)	792 (99.6)	153 (94.4)	< 0.001	0.003
Education (High school and above)	197 (24.8)	22 (13.6)	0.002	0.033
Number of days in hospital(Less than 7 days)	204 (25.7)	58 (35.8)	0.008	0.020
CRP	3.90 (1.60, 9.69)	5.87(2.14,19.52)	0.002	0.006
TC	4.31(3.45,5.24)	4.73(3.78,5.32)	0.012	< 0.001
HDL	1.18(1.04,1.40)	1.06(0.88,1.28)	< 0.001	< 0.001
LDL	2.25(1.71,2.85)	2.46(1.88,3.01)	0.006	0.010
ACEI/ARB/ARNI (NO)	373 (46.9)	60 (37.0)	0.021	0.009

CRP: C reactive protein; TC: Total cholesterol; HDL: high density lipoprotein; LDL: low density lipoprotein; ACEI/ARB/ARNI: angiotensin converting enzyme inhibitor/Angiotonin Receptor Blocker**/**Angiotensin receptor-neprilysin inhibitor.

**Table 2:** Common variables screened by three methods.

Methods	Common variables
Logistic regression	Discharge outcomes, Mode of admission, Communication skills, CRP, TC, HDL, LDL
LASSO regression	
random forest	

CRP: C reactive protein; TC: Total cholesterol; HDL: high density lipoprotein; LDL: low density lipoprotein.

### Model development

**Figure 3:**
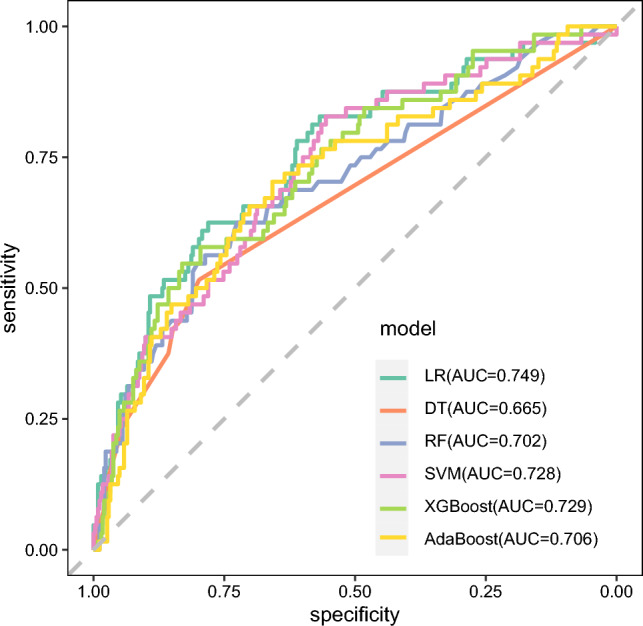
ROC for 6 machine learning algorithms. LR: logistic regression; DT: Decision Tree; RF: Random Forest; SVM: support vector machine; XGBoost: extreme gradient boosting; AdaBoost: Adaptive Boosting; AUC: area under the curve.

Based on 7 variables selected by three different methods, they are incorporated into the model development. Six ML models were constructed to predict the risk of the readmissions of NSTEMI patients after PCI (The statistical parameters of the six methods are shown in supplemental Table S5). Comparison of the AUC of six ML algorithms is shown in Fig. 3. As is shown in Table 3, the LR model achieved the highest AUC (0.749), outperforming the other five models. Additionally, the DT model had the lowest AUC (0.665). From the perspective of prediction accuracy, all models except SVM were relatively good, all above 65%, among which XGBoost performs best, followed by LR and DT. In terms of sensitivity and specificity, LR showed better performance. Therefore, based on the above four evaluation indicators, the LR algorithm is considered to be the most suitable for constructing prediction models.

**Table 3:** Prediction results of readmission of NSTEMI patients treated with PCI by 6 machine learning algorithms.

Model	AUC[95%CI]	*p*_value	Sensitivity	Specifty	Accuracy
LR	0.749[0.681–0.817]	*p* < 0.001	0.625	0.781	0.756
DT	0.665[0.597–0.733]	*p* < 0.001	0.516	0.798	0.754
RF	0.702[0.628–0.776]	*p* < 0.001	0.625	0.725	0.709
SVM	0.728[0.660–0.795]	*p* < 0.001	0.828	0.556	0.599
XGBoost	0.729[0.661–0.798]	*p* < 0.001	0.547	0.83	0.786
AdaBoost	0.706[0.633–0.779]	*p* < 0.001	0.703	0.658	0.665

LR: logistic regression; DT: Decision Tree; RF: Random Forest; SVM: support vector machine; XGBoost: extreme gradient boosting; AdaBoost: Adaptive Boosting; AUC: area under the curve; CI: confidence interval.

### The establishment of the prediction model nomogram

**Figure 4:**
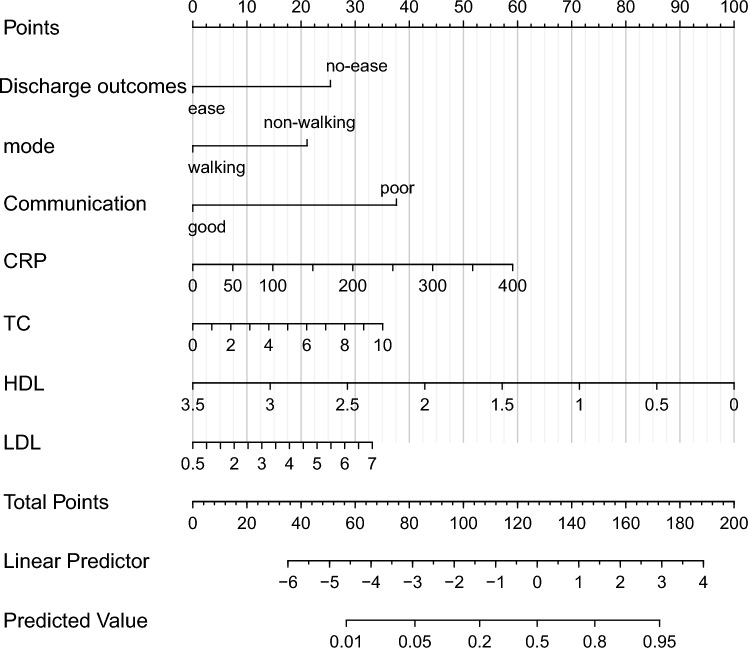
The nomogram of the LR model; CRP: C reactive protein; TC: Total cholesterol; HDL: high density lipoprotein; LDL: low density lipoprotein.

The LR algorithm, which is considered as the best ML method, was used to construct a prediction model. Furthermore, a nomogram was constructed (Fig. 4) according to its 7 predictive variables: discharge outcomes (ease, no-ease), admission mode (walking, non-walking), communication ability (good, poor), C reactive protein (CRP), total cholesterol (TC), high density lipoprotein (HDL), low density lipoprotein (LDL) (Fig. 4). (The supplementary material (example) provides examples of how to use nomogram to calculate scores and probabilities).The length of the lines in the figure reflects the contribution of the variables in the model to predicting the occurrence of the re-admission of NSTEMI patients after PCI. In addition, patients with better discharge outcomes during hospitalization, walking mode of admission, and better communication ability had a lower risk of readmission. Table 1 describes the included variables by training set using median (interquartile range) and frequency (composition ratio). In addition, the results of the univariate and multifactor regression are shown.

### Validation of predictive models

**Figure 5:**
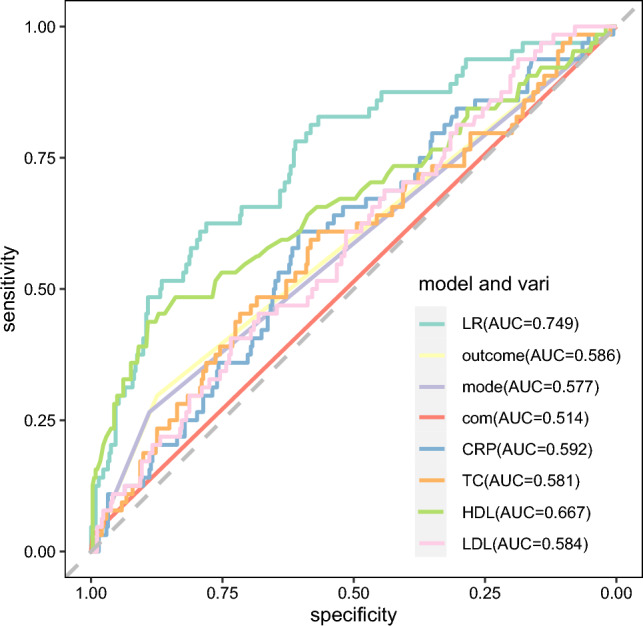
ROC analysis of optimal LR model and its 7 clinical variables. Outcome: discharge outcomes; mode: admission mode; com: communication ability; CRP: C reactive protein; TC: Total cholesterol; HDL: high density lipoprotein; LDL: low density lipoprotein.

**Figure 6:**
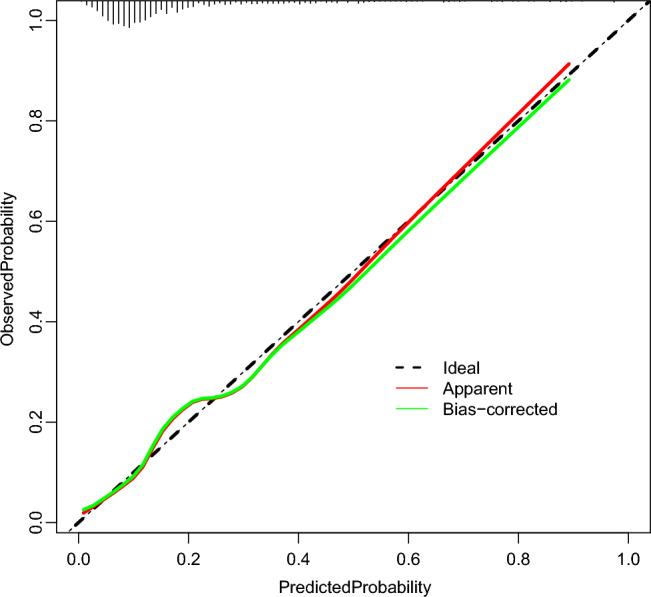
The calibration curve of the LR model. The ideal line indicates that the model prediction is exactly the same as the actual situation, which is the ideal situation. Apparent and bias.

**Figure 7:**
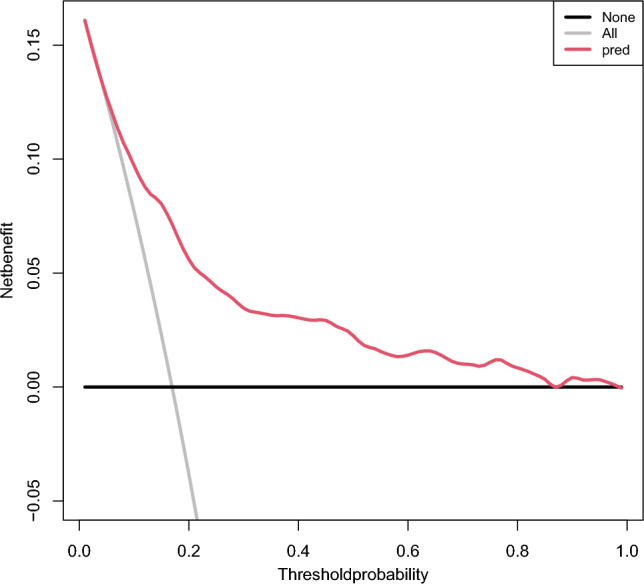
Decision curve analysis of readmission in NSTEMI patients within one year after PCI.Solid black line, assuming no readmissions have occurred in patients (indicated by none, i.e., horizontal line). The grey line indicates that all patients had readmissions (denoted by ALL, i.e., oblique line). The red line represents the calibration curve of the model.

Each variable is described by receiver ROC (Fig. 5). The results of ROC showed that the area under the curve of the nomogram model for predicting the re-admission of NSTEMI patients after PCI was 0.749, the sensitivity was 62.5%, and the specificity was 78.1% (Table 3), indicating that the prediction model had good discrimination and high prediction accuracy for the re-admission of NSTEMI patients after PCI. According to the model calibration curve analysis, the actual curve of the nomogram model predicting re-admission of NSTEMI patients after PCI was close to the ideal curve (Fig. 6), which basically fluctuated around the ideal curve, suggesting that the risk predicted by the model was in good agreement with the actual risk. In addition, this study evaluated the clinical practicability of the readmission risk prediction model for NSTEMI patients after PCI by drawing a clinical decision curve analysis (Fig. 7). The horizontal axis is the Threshold Probability, and the vertical axis is the Net Benefit after subtracting the benefits and harms. The black horizontal line represents the scenario where the model predicts that none of the patients will experience readmissions after PCI. In this case, the net clinical benefit rate is zero. The gray slash (All) indicates the negative slope of the net benefit rate when all patients in the predicted model have a readmissions outcome after PCI in the extreme case. The Readmissions prediction nomogram (red curve) is an actual clinical decision curve for readmissions after PCI. This curve is higher than the two extreme lines, indicating that this prediction model holds clinical practical value and potentially benefit patients.

**Figure 8:**
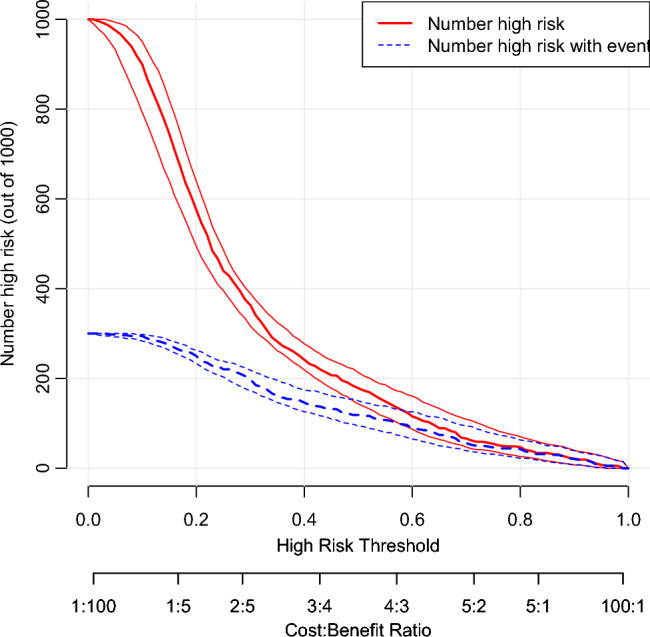
The clinical impact curve of the optimal prediction model is drawn based on the nomogram.

At the same time, the clinical impact curve further proves that the model has good clinical practicability (Fig. 8). In the figure, we can also see that the red line represents the number of people judged as high risk by the model under different probability thresholds. The blue lines represent the number of people the model judges to be at high risk, and the number of people who experience an outcome event at different probability thresholds. Therefore, Nomogram has good clinical application value.

### Comparison of prediction models

**Figure 9:**
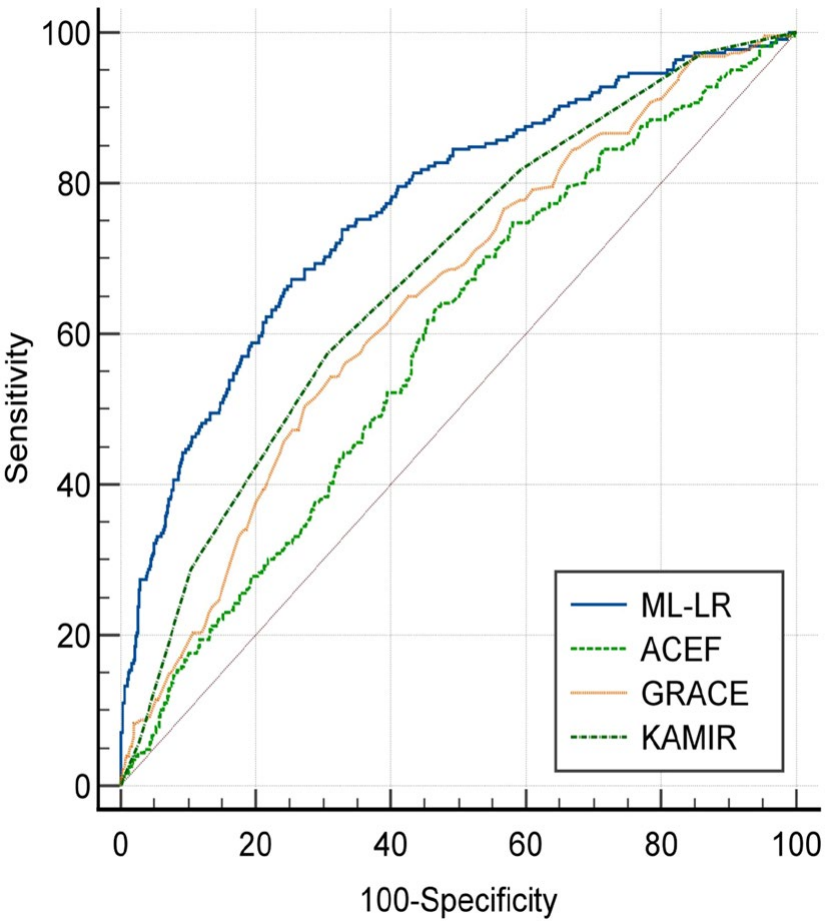
Comparison of ROC curves between the LR prediction model and the adjusted GRACE score, KAMIR score and ACEF score models.

To further evaluate the predictive power of this model, we compared it with previous scoring models. According to the DeLong test, The AUC of the adjusted GRACE score is 0.645(95% CI 0.619–0.670), the AUC of KAMIR score was 0.678 (95% CI 0.619). The AUC of ACEF score was 0.591 (95% CI 0.564–0.617) (Fig. 9). The prediction ability of the Nomogram model is better than that of the adjusted GRACE score (Z = 4.486, *P* < 0.001), KAMIR score (Z = 3.321, *P* < 0.001) and ACEF score (Z = 6.341, *P* < 0.001) (Table 4). (For details of each risk score, see Supplementary Materials—Risk Score Table).

**Table 4:** Comparison between the new model constructed by LR algorithm and other risk scoring models.

Model	AUC	(95%CI)	Z	*P* value
ML-LR	0.767	(0.743–0.789)	–	–
Adjusted GRACE	0.645	(0.619–0.670)	4.486	< 0.001
KAMIR	0.678	(0.653–0.703)	3.321	< 0.001
ACEF	0.591	(0.564–0.617)	6.341	< 0.001

Adjusted GRACE: adjusted global registry of acute coronary events risk score; KAMIR: The Korea Acute Myocardial Infarction Registry risk score; ACEF: The Age, creatinine, and ejection fraction score.

## Discussion

Cardiovascular disease has become the leading cause of death worldwide. Acute myocardial infarction is the most serious type of coronary heart disease due to its high incidence, sudden onset, rapid development and high mortality. In recent years, the proportion of NSTEMI in myocardial infarction has increased year by year^23^, becoming the main type of myocardial infarction. This seriously interferes with patients' daily life and even endangers their lives^24,25^, posing considerable challenges to social medical care. At present, PCI is the main way to treat myocardial infarction by dredging blocked coronary vessels and improving ischemic symptoms. However, patients usually may be readmitted to hospital due to recurrent myocardial infarction and myocardial infarction complications after a myocardial infarction. Therefore, it is of great significance to effectively identify high-risk groups and reduce the readmission rate of such patients as much as possible.

In recent years, based on the rapid development of artificial intelligence technology, it is also shining brightly in various industries. In medicine, machine learning, a branch of artificial intelligence science, is in full swing in clinical practice, using its characteristics of efficient and accurate processing of high-dimensional interactive information in large data sets. Common features in large datasets to identify complex, multidimensional, and non-linear relationships between clinical features to predict various outcomes have shown unique clinical applications in many medical fields, including oncology^8^ and neurology^26^. Remarkable achievements have also been made in the cardiovascular field, such as D'Ascenzo F's Artificial Intelligence Risk Prediction after acute Coronary Syndrome (PRAISE), etc.^27^.

At present, there are few predictive models for NSTEMI patients' readmissions after PCI. Therefore, this study based on multiple ML methods screened the risk factors for NSTEMI patients' readmissions after PCI and established predictive models. A total of 1576 patients diagnosed with NSTEMI and treated by PCI were selected. After collecting their clinical data and screening, 1363 patients were finally included and divided into the training set and validation set according to the ratio of 7:3 according to the time sequence. In the training set and validation set, the patients were divided into the readmission group and the non-readmission group according to whether they were readmitted (Fig. 1). In order to ensure the representativeness of the variables, three complementary methods were used to screen the variables, making them more conducive to identifying the characteristic variables closely related to the prediction of outcome events. After selection, seven factors affecting the re-admission of NSTEMI patients after PCI were found. These included discharge outcomes (ease, no-ease), admission mode (walking, non-walking), communication ability (good, poor), CRP, TC, HDL, LDL. Six ML algorithm, LR, DT, RF, SVM, XGBoost and AdaBoost, were used to develop the model, and the prediction efficiency of each model was evaluated by AUC, accuracy, sensitivity and specificity. After comparison, the prediction model constructed by LR algorithm was 0.749, with a sensitivity of 62.5% and a specificity of 78.1%. The accuracy was 75.6%, which proved that the model had good discrimination. Therefore, the LR algorithm was finally selected and a nomogram for predicting the risk of readmission in NSTEMI patients after PCI was established based on it. In order to further evaluate the accuracy of the model, the calibration curve was used. As shown in the Fig. 6, the performance curve of the model had good consistency with the calibration curve, basically fluctuated around the ideal curve, and the prediction model had good accuracy. To assess the clinical risk prediction model is practical, so as to draw the curve of clinical decision and clinical impact curve were analyzed, the curve shows that the prediction model has clinical practical value and can potentially benefit patients.

Previous studies have shown that models constructed by machine learning methods are better than traditional risk scoring models. For example, Kwon JM's study proposed that models constructed by deep learning in machine learning algorithms were better than GRACE risk scoring models in predicting in-hospital mortality of AMI patients (AUC: 0.905 vs 0.851, *P* < 0.001);Qiu H also proposed that the risk model of acute kidney injury in patients with acute ST-segment elevation myocardial infarction after PCI constructed based on LASSO regression was superior to the Mehran score 2 scoring model (AUC: 0.840 vs 0.674, *P* < 0.001).Therefore, to further verify the performance of this model, we will compare it with common clinical risk assessment models, including adjusted GRACE score, the KAMIR score and the ACEF score. By comparison, The prediction ability of the Normograph model is better than that of the adjusted GRACE score (AUC: 0.767 vs 0.645, Z = 4.486, *P* < 0.001), KAMIR score (AUC: 0.767 vs 0.678, Z = 3.321, *P* < 0.001) and ACEF score (AUC: 0.767 vs 0.591, Z = 6.341, *P* < 0.001) (Table 4). Therefore, the model established by us has good prediction ability.

In this study, we comprehensively included relevant indicators of patient hospitalization. These indicators encompassed basic admission information such as admission pathway (emergency or outpatient), admission mode (walking or non-walking), admission consciousness (awake or not awake), admission communication ability (good or poor), sleep status (normal or abnormal), and more. This level of details is very rare in previous studies. We believe that the detailed collection of patient data is conducive to the comprehensive evaluation of patients. In our study, admission mode (walking or non-walking) and admission communication ability (good or poor) were independent risk factors for readmissions in NSTEMI patients after PCI. This is also in line with our conventional cognition, non-walking admission and poor admission communication skills indicate poor general condition of patients, which can also indicate that the heart function of NSTEMI patients after PCI is still poor after discharge, thus affecting the re-admission rate of patients.

For the discharge status of patients, we also focused on their discharge outcome (ease or no-ease). For normal low-risk patients, early discharge after PCI is a safety strategy^28^, and we will require them to be discharged quickly after their condition is stable, that is, they will be discharged after improvement. However, in our research, it was found that the proportion of discharge outcomes that were not improved in the readmission group was significantly higher than that in the non-readmission group, suggesting that discharge outcomes were a characteristic variable affecting readmission, which may be due to the fact that such patients did not fully recover during hospitalization and often had vascular, bleeding or cardiac complications before discharge^29^. More medication and longer hospital stay are required, so early discharge is more likely to be re-admission. Therefore, it is required to provide steady treatment during hospitalization, rather than discharging patients without improvement, as this can play a role in preventing readmissions^30,31^

For the inflammatory indicators of patients, we focused on CRP, because studies have shown that inflammatory factors have certain value in predicting the prognosis of acute myocardial infarction^32^, and the risk of adverse prognostic events after PCI is positively correlated with CRP levels^33^.In this study, CRP levels in the readmitted group were significantly higher than those in the non-readmitted group, which also confirmed the study of Carrero JJ et al.^32^. CRP belongs to the acute phase protein, which is mainly synthesized by the liver. When infection or tissue damage occurs, the synthesis of liver will increase significantly, and the level of CRP will also increase significantly. The more significant the structural changes of the heart, the lower the cardiac function of the patient, indicating that CRP is related to the occurrence of ventricular remodeling^34^. Therefore, for NSTEMI patients after PCI, we should pay close attention to their CRP index to reduce the readmission rate.

In this study, TC, LDL, and HDL were all considered risk factors for readmissions of NSTEMI patients after PCI, and the readmission group had higher TC, higher LDL, and lower HDL than the non-readmission group. It also accords with our normal cognition, namely, the core of coronary heart disease pathogenesis have dyslipidemia. In the 2019 European Society of Cardiology (European Society of Cardiology, ESC)/European Atherosclerosis Society (EAS) lipid guidelines^35^ and the 2018 Chinese Guidelines for the Diagnosis and Treatment of Stable Coronary Heart Disease^36^ emphasize that the risk of coronary heart disease progression is positively associated with blood lipids, particularly low-density lipoprotein (LDL). Relevant lipid indicators such as total cholesterol (TC), triglyceride (TG), low-density lipoprotein (LDL) and high-density lipoprotein (HDL) have always been considered as important tools for the prevention and treatment of coronary heart disease in clinical practice, while high TC, high LDL and low HDL are independent risk factors for cardiovascular disease^37^. TC measurement is a useful screening index for the detection of high-risk groups of coronary artery disease. High cholesterol is a risk factor for coronary artery disease, and it is also the main substance that promotes arteriosclerosis, which has an obvious effect on the readmissions of patients. HDL can carry cholesterol in the surrounding tissues and convert it into bile acids or directly expel it through the intestine^38^, this process lead to decreased cholesterol levels and lowers the risk of readmissions, which further confirms the study of Lin T et al.^39^. The study further confirmed that HDL is a protective factor for atherosclerosis, and that its level can reflect coronary artery lesions, which in turn affects the readmission rate. The effect of LDL is opposite to that of HDL. Endothelial function is impaired in the early stage of atherosclerosis, and the phagocytosis of oxidized LDL by monocyte-macrophages to form foam cells is the core link of atherosclerosis^40^. Observational studies and randomized controlled trials have proved that LDL is one of the important factors affecting the prognosis and readmissions of coronary heart disease^41,42^. Therefore, lowering LDL has a clear benefit in reducing readmission rates.

This study has the following advantages: First, it focus on the analysis of NSTEMI, the main type of myocardial infarction, which is rare in current studies. In addition, representative variables are selected by various methods, predictive models are established, and the models are compared. Second, the study includes many variables, and incorporates various indicators of patients' hospitalization, including both admission and discharge data. This comprehensive approach allows for the identification of previously unknown factors that influence readmission rates. Third, a concise and clear column chart was established, and the relevant indicators were easy to obtain and had good clinical practicability, which was conducive to using limited medical resources for patients with high risk of readmission. And this research has important significance in reducing readmission rate, medical burden and social and economic burden of patients.

## Limitations

This study has certain limitations. First of all, it is a single-center study, and although it has been divided into training set and verification set according to time order, no external verification has been conducted, and selection bias still exists. For example, age has been shown to be a risk factor for readmission after PCI in a number of large foreign retrospective studies^43,44^, but the age difference in this study was not statistically significant. Some elderly patients with heavy disease burden may choose local hospitals to seek treatment when they have postoperative discomfort, resulting in bias in readmission rate. Therefore, further multi-center and large sample research is still needed in the future. Second, the included indicators are still incomplete, and the relevant indicators of electrocardiogram have not been comprehensively evaluated, which may have an impact on the readmitted rate. The relevant indicators can be included for further analysis in the future, so as to make the model more effective in clinical evaluation. In addition, as a prognostic study of patients readmitted after discharge, no relevant survival analysis was available. If patients can have a longer follow-up period, it is believed that the judgment of patient readmission will be more favorable. Third, the sensitivity of the model is relatively low, although it is still greater than 60%. However, it is important to highlight that the accuracy and specificity of the model are well-balanced. This indicates that the majority of readmissions can still be accurately predicted, which is of great significance for the rational use of medical resources. Fourth, not all machine model methods are used (such as elastic network, neural network, etc.), but six most commonly used modeling methods are still selected and compared. The results obtained can still be considered robust and reasonable.

## Conclusions

This study employed three variable screening methods and four evaluation indicators to establish the superiority of the LR prediction model. Additionally, the nomogram provided a visual assessment of readmission probability for NSTEMI patients, offering valuable guidance for readmission risk assessment after PCI. Moreover, it may offer valuable insights into the study of myocardial infarction readmission rates among Asian populations.

### Supplementary Information

Supplementary Information 1.

Supplementary Information 2.

Supplementary Information 3.

Supplementary Information 4.

Supplementary Information 5.

Supplementary Information 6.

Supplementary Information 7.

Supplementary Information 8.

Supplementary Information 9.

Supplementary Information 10.

Supplementary Information 11.

## Data Availability

The datasets generated and/or analysed during the current study are not publicly available due to privacy or ethical restrictions but are available from the corresponding author on reasonable request.
