# Supplementary material for: Development and validation of a machine learning-based readmission risk prediction model for non-ST elevation myocardial infarction patients after percutaneous coronary intervention
Source: Sci Rep. 2024 Jun 11;14:13393. doi: 10.1038/s41598-024-64048-x (PMC11166920; doi:10.1038/s41598-024-64048-x)
Supplement: Supplementary file 1 — Supplementary Information 1. [file 41598_2024_64048_MOESM1_ESM.docx]

**Supplementary Materials**

**Example of risk calculation**

For example, an elderly patient with chest pain was wheeled into the ward of the inpatient department, NSTEMI was considered for PCI treatment, and his communication ability was poor at admission. The complete examination during hospitalization indicated CRP 2.93mg/L, total cholesterol(TC) 4.13mmol/L, HDL 1.83mmol/L, LDL 2.88mmol/L, and he was forced to be discharged after 4 days of hospitalization due to his own factors.

Calculate the patient's risk of readmission:

Non-walking admission (wheelchair) :25 points

Poor communication skills:21points

Discharge outcome(no-ease):37.5 points

CRP(2.93):1 points

TC(4.13):15 points

HDL(1.83):47.5 points

LDL(2.88):12 points

Total:25+21+37.5+1+15+47.5+12=159 points,and the patient's risk of readmission was 87.5%
