# Supplementary material for: Development and validation of a machine learning-based readmission risk prediction model for non-ST elevation myocardial infarction patients after percutaneous coronary intervention
Source: Sci Rep. 2024 Jun 11;14:13393. doi: 10.1038/s41598-024-64048-x (PMC11166920; doi:10.1038/s41598-024-64048-x)
Supplement: Supplementary file 2 — Supplementary Information 2. [file 41598_2024_64048_MOESM2_ESM.docx]

**Figure legends:**

**Figure 1.** Study design.NSTEMI: Non-ST Elevation Myocardial Infarction; PCI:

Percutaneous Coronary Intervention; LASSO: least absolute shrinkage and selection

operator ; LR: logistic regression ; DT: Decision Tree ; RF: Random Forest ; SVM:

support vector machine ; XGBoost: extreme gradient boosting ; AdaBoost: Adaptive

Boosting ; AUC: area under the curve.

**Figure 2.** Venn plots reflect the number of results for the three variable screening methods; The overlapping part is the seven variables selected, Logistic regression (LR) non-overlapping part has 4 variables, Random Forest (RF) non-overlapping section has 13 variables, Least absolute shrinkage and selection operator (LASSO) has 28 variables in the non-overlapping section.

**Figure 3.** ROC for 6 machine learning algorithms. LR: logistic regression; DT: Decision Tree; RF: Random Forest; SVM: support vector machine; XGBoost: extreme gradient boosting; AdaBoost: Adaptive Boosting; AUC: area under the curve.

**Figure 4.** The nomogram of the LR model; CRP: C reactive protein; TC: Total cholesterol; HDL: high density lipoprotein; LDL: low density lipoprotein

**Figure 5.** ROC analysis of optimal LR model and its 7 clinical variables.

outcome: discharge outcomes; mode: admission mode; com: communication

ability; CRP: C reactive protein; TC: Total cholesterol; HDL: high density

lipoprotein; LDL: low density lipoprotein.

**Figure 6.** The calibration curve of the LR model. The ideal line indicates that the model prediction is exactly the same as the actual situation, which is the ideal situation. Apparent and bias‐corrected lines indicate the prediction performance of the LR model.

**Figure 7.**Decision curve analysis of readmission in NSTEMI patients within one year

after PCI.Solid black line, assuming no re-admissions have occurred in patients (indicated by none, i.e., horizontal line). The grey line indicates that All patients had re-admissions (denoted by ALL, i.e., oblique line).The red line represents the calibration curve of the model.

**Figure 8.**The clinical impact curve of the optimal prediction model is drawn

based on the nomogram.

**Figure 9.**Comparison of ROC curves between the LR prediction model and the adjusted GRACE score, KAMIR score and ACEF score models.
