# Supplementary material for: Development and validation of a machine learning-based readmission risk prediction model for non-ST elevation myocardial infarction patients after percutaneous coronary intervention
Source: Sci Rep. 2024 Jun 11;14:13393. doi: 10.1038/s41598-024-64048-x (PMC11166920; doi:10.1038/s41598-024-64048-x)
Supplement: Supplementary file 4 — Supplementary Information 4. [file 41598_2024_64048_MOESM4_ESM.pdf]

Figure S2

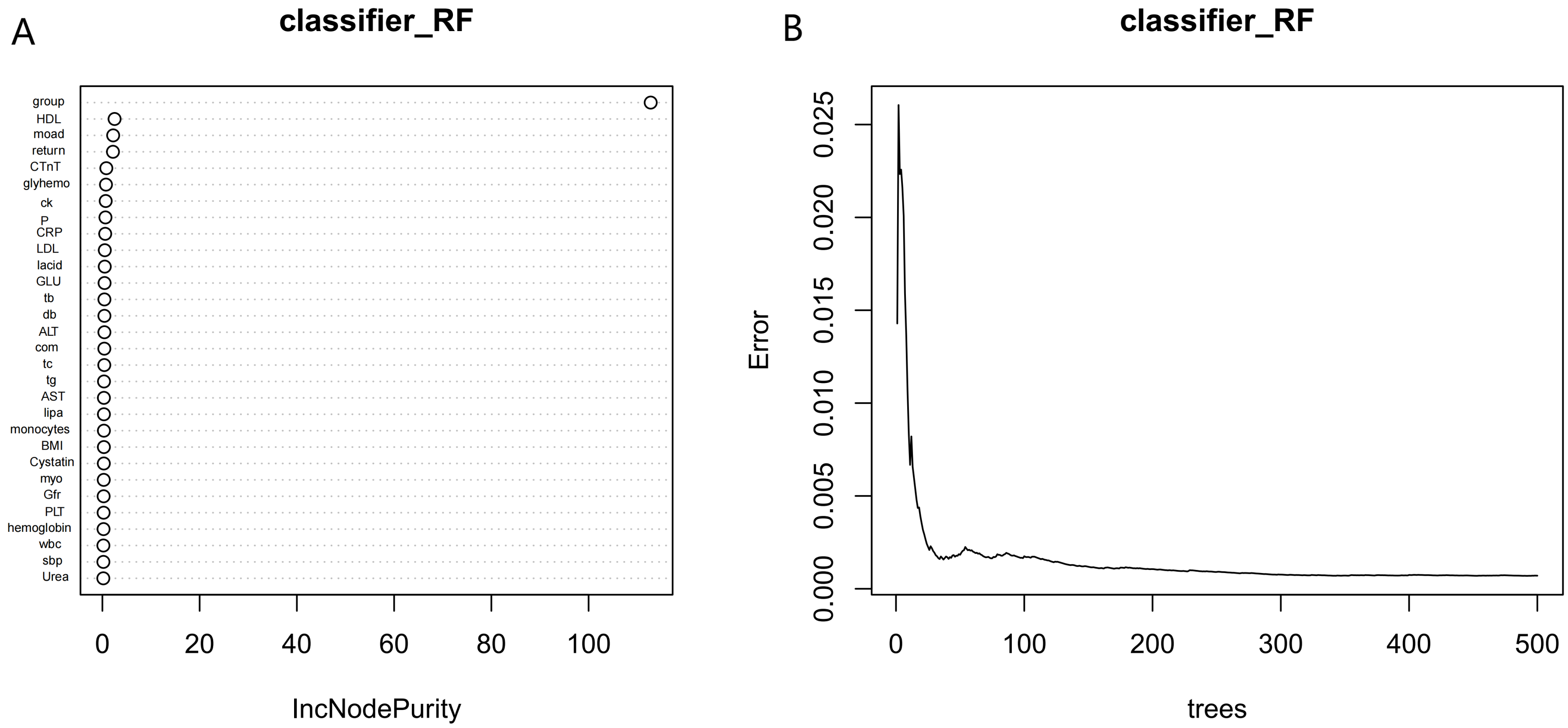

Figure S2. Feature selection was performed using random forest (RF) ;**(A)** The random forest method was used to sort variables according to their importance and select the top 20 variables. **(B)** Random forest error rate graph. As the trees increase, the error gradually decreases, and when the trees=100, the error becomes stable.
