# Supplementary material for: Development and validation of a machine learning-based readmission risk prediction model for non-ST elevation myocardial infarction patients after percutaneous coronary intervention
Source: Sci Rep. 2024 Jun 11;14:13393. doi: 10.1038/s41598-024-64048-x (PMC11166920; doi:10.1038/s41598-024-64048-x)
Supplement: Supplementary file 6 — Supplementary Information 6. [file 41598_2024_64048_MOESM6_ESM.docx]

**S Figure Legends:**

**Figure S1.** Feature selection was performed using the least absolute shrinkage and selection operator regression (LASSO) .**(A)** Variable selection via LASSO. As Lambda increases, the coefficients for some variables decrease until they reach zero. **(B)** LASSO regression model cross-validation plot. Draw a vertical line at the optimum with the minimum criterion and 1se of the minimum criterion.

When λ = 0. 01114426, we get 35 variables for further analysis.

**Figure S2.** Feature selection was performed using random forest (RF) ;**(A)** The random forest method was used to sort variables according to their importance and select the top 20 variables. **(B)** Random forest error rate graph. As the trees increase, the error gradually decreases, and when the trees=100, the error becomes stable.
