# Supplementary material for: Development and validation of a machine learning-based readmission risk prediction model for non-ST elevation myocardial infarction patients after percutaneous coronary intervention
Source: Sci Rep. 2024 Jun 11;14:13393. doi: 10.1038/s41598-024-64048-x (PMC11166920; doi:10.1038/s41598-024-64048-x)
Supplement: Supplementary file 7 — Supplementary Information 7. [file 41598_2024_64048_MOESM7_ESM.docx]

**S1 Baseline characteristics for all variables**

| Readmission of patients with NSTEMI after PCI surgery (total) | | | |
| --- | --- | --- | --- |
| variable | non-readmission(N=1137) | re-admission (N=226) | P |
| Sex（female）(%) | 305（26.8） | 73（32.3） | 0.093 |
| Ethnicity (Han)(%) | 1137（100） | 224（99.1） | 0.027 |
| BMI | 23.61±2.95 | 23.61±3.00 | 0.996 |
| Age | 64.50±11.34 | 69.23±10.76 | <0.001 |
| Admission room(%) |  |  | 0.216 |
| emergency | 163(14.3) | 24(10.6) |  |
| Outpatient | 949(83.5) | 199(88.1) |  |
| other | 25(2.2) | 3(1.3) |  |
| Discharge outcomes(%) |  |  | <0.001 |
| Ease | 1054(92.7) | 169（74.8） |  |
| Non-ease | 83（7.3） | 57（25.2） |  |
| medicare(%) |  |  | 0.144 |
| No | 79（6.9） | 22（9.7） |  |
| Yes | 1058（93.1） | 204（90.3） |  |
| Education(%) |  |  | <0.001 |
| High school or less | 847（74.5） | 195（86.3） |  |
| High school and above | 290（25.5） | 31（13.7） |  |
| marriage(%) |  |  | 0.149 |
| married | 1087（95.6） | 211（93.4） |  |
| unmarried | 50（4.4） | 15（6.6） |  |
| Systolic blood pressure(M (P25, P75)) | 128（115，146） | 130（116，150.25） | 0.250 |
| Diastolic blood pressure | 78（69，87） | 77（69，87） | 0.408 |
| heart rate | 76（68，85） | 76（68.75，83） | 0.821 |
| body temperature | 36.6（36.5，36.7） | 36.6（36.5，36.7） | 0.266 |
| Number of breaths | 20（19，21） | 20（19，21） | 0.135 |
| mode(%) |  |  | <0.001 |
| Walking | 1007（88.6） | 153（66.7） |  |
| No-walking | 130（11.4） | 73（32.3） |  |
| awareness(%) |  |  | 0.107 |
| awake | 1116（98.2） | 218（96.5） |  |
| Non-awake | 21（1.8） | 8（3.5） |  |
| Communication skills(%) |  |  | <0.001 |
| Good | 1133（99.6） | 215（95.1） |  |
| Poor | 4（0.4） | 11（4.9） |  |
| Ejection fraction(%) |  |  | 0.047 |
| ≥50% | 979(86.1) | 183(81.0) |  |
| <50% | 158(13.9) | 43(19.0) |  |
| diabetes(%) |  |  | 0.001 |
| No | 706（62.1） | 114（50.4） |  |
| Yes | 431（37.9） | 112（49.6） |  |
| hypertension(%) |  |  | 0.065 |
| No | 462（40.6） | 77（34.1） |  |
| Yes | 675（59.4） | 149（65.9） |  |
| Stroke(%) |  |  | <0.001 |
| No | 881（77.5） | 148（65.5） |  |
| Yes | 256（22.5） | 78（34.5） |  |
| Peripheral vascular lesions(%) |  |  | 0.186 |
| No | 456（40.1） | 80（35.4） |  |
| Yes | 681（59.9） | 146（64.6） |  |
| pneumonia(%) |  |  | 0.324 |
| No | 694（61.0） | 130（57.5） |  |
| Yes | 443（39.0） | 96（42.5） |  |
| Changes in the structure of the heart(%) |  |  | 0.002 |
| No | 733（64.5） | 121（53.5） |  |
| Yes | 404（35.5） | 105（46.5） |  |
| Rhythm(%) |  |  | 0.726 |
| Sinus | 823（72.4） | 161（71.2） |  |
| Non-sinus | 314（27.6） | 65（28.8） |  |
| Myocardial bridge(%) |  |  | 0.027 |
| No | 1106（97.3） | 218（96.5） |  |
| Yes | 31（2.7） | 8（3.5） |  |
| CTO(%) |  |  | 0.027 |
| No | 1108（97.4） | 214（94.7） |  |
| Yes | 29（2.6） | 12（5.3） |  |
| Gastrointestinal bleeding(%) |  |  | 0.224 |
| No | 1062（93.4） | 206（91.2） |  |
| Yes | 75（6.6） | 20（8.8） |  |
| heart failure(%) |  |  | 0.269 |
| No | 950（83.6） | 182（80.5） |  |
| Yes | 187（16.4） | 44（19.5） |  |
| Grading of cardiac function(%) |  |  | 0.002 |
| 1-2 | 939（82.6） | 167（73.9） |  |
| 3-4 | 198（17.4） | 59（26.1） |  |
| Diffuse coronary changes(%) |  |  | 0.176 |
| No | 934（82.1） | 177（78.3） |  |
| Yes | 203（17.9） | 49（21.7） |  |
| Pathological Q waves(%) |  |  | 0.167 |
| No | 990（87.1） | 189（83.6） |  |
| Yes | 147（12.9） | 37（16.4） |  |
| Number of diseased blood vessels(%) |  |  | 0.030 |
| Less than 3 sticks | 527(46.4) | 87(38.5) |  |
| 3 or more | 610（53.6） | 139（61.5） |  |
| Criminal coronary vessels(%) |  |  | 0.600 |
| Single | 854（75.1） | 166（73.5） |  |
| Multi-branch | 283（24.9） | 60（26.5） |  |
| Drinking(%) |  |  | 0.450 |
| Yes | 443（39.0） | 82（36.3） |  |
| No | 694（61.0） | 144（63.7） |  |
| Smoking(%) |  |  | 0.156 |
| Yes | 602（52.9） | 108（47.8） |  |
| No | 535（47.1） | 118（52.2） |  |
| Number of days in hospital(%) |  |  | 0.001 |
| Less than 7 days | 287(25.2) | 82(36.3) |  |
| Greater than or equal to 7 days | 850(74.8) | 144(63.7) |  |
| Sleeping(%) |  |  | 0.403 |
| normal | 487（42.8） | 90（39.8） |  |
| abnormal | 650（57.2） | 136（60.2） |  |
| TIMI blood flow(%) |  |  | 0.018 |
| 2 | 15（1.3） | 8（3.5） |  |
| 3 | 1122(98.7) | 218（96.5） |  |
| Number of stents | 1（1，2） | 1（1，2） | 0.076 |
| Rotary grinding(%) |  |  | 0.417 |
| No | 1077（94.7） | 217（96） |  |
| Yes | 60（5.3） | 9（4） |  |
| Intraoperative hypotension(%) |  |  | 0.201 |
| No | 1049（92.3） | 214（94.7） |  |
| Yes | 88（7.7） | 12（5.3） |  |
| Intraoperative arrhythmia(%) |  |  | 0.393 |
| No | 1055（92.8） | 206（91.2） |  |
| Yes | 82（7.2） | 20（8.8） |  |
| Intraoperative vascular ultrasound(%) |  |  | 0.098 |
| No | 1074（94.5） | 207（91.6） |  |
| Yes | 63（5.5） | 19（8.4） |  |
| red blood cells | 4.35±0.67 | 4.33±0.70 | 0.655 |
| Hematocrit | 0.4±0.06 | 0.4±0.07 | 0.398 |
| platelet | 184.16±65.57 | 180.58±68.99 | 0.458 |
| lymphocyte | 1.53±0.64 | 1.45±0.64 | 0.068 |
| monocyte | 0.50±0.23 | 0.51±0.24 | 0.401 |
| white blood cell | 7.85±3.04 | 7.66±2.98 | 0.404 |
| Neutrophils | 5.63±2.97 | 5.51±2.83 | 0.571 |
| Neutrophil ratio | 0.70±0.11 | 0.70±0.11 | 0.921 |
| haemoglobin | 128.43±21.36 | 122.66±21.57 | <0.001 |
| INR | 1.07±0.29 | 1.09±0.27 | 0.365 |
| PLR | 3.29（2.3，5.19） | 3.33（2.33，5.78） | 0.350 |
| NLR | 121.00（89.25，171.68） | 126.08（94.23，174.18） | 0.440 |
| D-dimer | 0.84（0.62，1.25） | 0.88（0.61，1.31） | 0.549 |
| D-dimer peak | 0.98（0.66，1.52） | 0.98（0.68，1.61） | 0.534 |
| CRP | 3.97（1.69，8.88） | 6.23（2.5，12.75） | <0.001 |
| myoglobin | 51.53（32.54，94.14） | 53.36（34.55，88.14） | 0.591 |
| creatine kinase | 3.21（1.71，11.47） | 3.76（2.32，8.01） | 0.119 |
| Troponin T | 0.24（0.09，1.17） | 0.52（0.22，1.62） | <0.001 |
| BNP | 567(178.46,1542.00） | 632.35(232.13,1378.46） | 0.223 |
| homocysteine | 16.06±7.29 | 16.39±8.97 | 0.549 |
| Fasting glucose | 8.02±3.35 | 9.10±4.54 | 0.001 |
| creatinine | 76.6（65,94.15） | 82.0(63.63,104.02) | 0.113 |
| Glomerular filtration rate | 83.57(67.10,96.26) | 77.19(57.34,97.62) | 0.026 |
| urea | 5.8（4.56,7.59） | 6.31（4.66,8.16） | 0.038 |
| Cystatin | 1.10(0.94,1.36) | 1.22(1.01,1.61) | <0.001 |
| uric acid | 348.14(289.65,425.95) | 356.64(293.74,427.97) | 0.680 |
| lactic acid | 1.60(1.18,2.22) | 1.93(1.44,2.59) | <0.001 |
| phosphorus | 0.95(0.86,1.08) | 0.95(0.86,1.07) | 0.434 |
| magnesium | 0.84(0.79,0.90) | 0.84(0.79,0.90) | 0.697 |
| potassium | 3.91(3.62,4.26) | 3.90(3.60,4.23) | 0.483 |
| sodium | 138.10(136.00,141.65) | 138.92(136.64,141.59) | 0.536 |
| calcium | 2.28(2.19,2.37) | 2.27(2.18,2.35) | 0.132 |
| Total bilirubin | 13.2(9.69,17.00) | 11.87(9.2,16.46) | 0.014 |
| Direct bilirubin | 3.24(2.3,4.64) | 2.93(2.40,3.90) | 0.255 |
| albumin | 38.5(35.80,41.18) | 37.5(35.00,40.53) | 0.005 |
| ALT | 17.5(10.00,30.75) | 16.63(11.08,26.75) | 0.923 |
| Lipoprotein A | 199.7(90.95,375.96) | 193.74(87.59,367.775) | 0.827 |
| TC | 4.28(3.56,5.19) | 4.71(3.81,5.42) | 0.001 |
| TG | 1.46(1.08,2.01) | 1.69(1.32,2.19) | <0.001 |
| Glycated hemoglobin | 7.21(6.5,8.09) | 7.60(6.57,8.97) | 0.003 |
| AST | 30.00(22.5,41.5) | 28.85(23.95,39.00) | 0.847 |
| HDL | 1.19(1.04,1.40) | 1.06(0.87,1.29) | <0.001 |
| LDL | 2.23(1.73,2.82) | 2.44(1.91,3.03) | <0.001 |
| heparin(%) |  |  | 0.582 |
| No | 8(0.7) | 3(1.3) |  |
| Yes | 1129(99.3) | 223(98.7) |  |
| Antiplatelet drug types(%) |  |  | 0.239 |
| 1 | 8(0.7) | 4(1.8) |  |
| 2 | 1129(99.3) | 222(98.2) |  |
| Statins(%) |  |  | 1.000 |
| No | 6(0.5) | 1(0.4) |  |
| Yes | 1131(99.5) | 225(99.6) |  |
| Proton pump inhibitors(%) |  |  | 0.616 |
| No | 81(7.1) | 14(6.2) |  |
| Yes | 1056(92.9) | 212(93.8) |  |
| ACEI/ARB/ARNI (%) |  |  | 0.002 |
| No | 535(47.1) | 81(35.8) |  |
| Yes | 602(52.9) | 145(64.2) |  |
| B-blockers(%) |  |  | 0.003 |
| No | 284(25.0) | 36(15.9) |  |
| Yes | 853(75.0) | 190(84.1) |  |
| CCB(%) |  |  | 0.674 |
| No | 756(66.5) | 147(65.0) |  |
| Yes | 381(33.5) | 79(35.0) |  |
| Nitrates(%) |  |  | 0.346 |
| No | 152(13.4) | 25(11.1) |  |
| Yes | 985(86.6) | 201(88.9) |  |
| Hypoglycemic agents(%) |  |  | 0.017 |
| No | 829(72.9) | 147(65.0) |  |
| Yes | 308(27.1) | 79(35.0) |  |
