# Supplementary material for: Development and validation of a machine learning-based readmission risk prediction model for non-ST elevation myocardial infarction patients after percutaneous coronary intervention
Source: Sci Rep. 2024 Jun 11;14:13393. doi: 10.1038/s41598-024-64048-x (PMC11166920; doi:10.1038/s41598-024-64048-x)
Supplement: Supplementary file 8 — Supplementary Information 8. [file 41598_2024_64048_MOESM8_ESM.docx]

**S2 Baseline characteristics for all variables**

| Readmission of patients with NSTEMI after PCI surgery (training cohort) | | | |
| --- | --- | --- | --- |
| variable | non-readmission(N=795) | re-admission (N=162) | P |
| Sex（female） | 210（26.4） | 48（29.6） | 0.401 |
| Ethnicity (Han) | 795（100） | 161（99.4） | 0.169 |
| BMI | 23.59±2.93 | 23.73±2.99 | 0.568 |
| Age | 64.05±11.39 | 68.74±10.55 | <0.001 |
| Admission room |  |  | 0.133 |
| emergency | 120（15.1） | 17（10.5） |  |
| Outpatient | 652（82） | 143（88.3） |  |
| other | 23（2.9） | 2（1.2） |  |
| Discharge outcomes |  |  | <0.001 |
| Ease | 755（95.0） | 124（76.5） |  |
| Non-ease | 40（5.0） | 38（23.5） |  |
| medicare |  |  | 0.237 |
| No | 57（7.2） | 16（9.9） |  |
| Yes | 738（92.8） | 146（90.1） |  |
| Education |  |  | 0.002 |
| High school or less | 598（75.2） | 140（86.4） |  |
| High school and above | 197（24.8） | 22（13.6） |  |
| marriage |  |  | 0.258 |
| married | 762（95.8） | 152（93.8） |  |
| unmarried | 33（4.2） | 10（6.2） |  |
| Systolic blood pressure | 130（115，147） | 130（117，150） | 0.459 |
| Diastolic blood pressure | 78（70，88） | 77（69，88） | 0.558 |
| heart rate | 76（68，85） | 76（70，83） | 0.878 |
| body temperature | 36.6(36.5,36.7) | 36.6(36.5,36.7) | 0.148 |
| Number of breaths | 20(19,21) | 20(19,21) | 0.306 |
| mode |  |  | <0.001 |
| Walking | 703（88.4） | 106（65.4） |  |
| No-walking | 92（11.6） | 56（34.6） |  |
| awareness |  |  | 0.215 |
| awake | 782（98.4） | 157（96.9） |  |
| Non-awake | 13（1.6） | 5（3.1） |  |
| Communication skills |  |  | <0.001 |
| Good | 792（99.6） | 153（94.4） |  |
| Poor | 3（0.4） | 9（5.6） |  |
| Ejection fraction |  |  | 0.062 |
| ≥50% | 692（87） | 132（81.5） |  |
| <50% | 103（13） | 30（18.5） |  |
| diabetes |  |  | 0.009 |
| No | 490（61.6） | 82（50.6） |  |
| Yes | 305（38.4） | 80（49.4） |  |
| hypertension |  |  | 0.133 |
| No | 305（38.4） | 52（32.1） |  |
| Yes | 490（61.6） | 110（67.9） |  |
| Stroke |  |  | 0.004 |
| No | 610（76.7） | 107（66） |  |
| Yes | 185（23.3） | 55（34） |  |
| Peripheral vascular lesions |  |  | 0.265 |
| No | 317（39.9） | 57（35.2） |  |
| Yes | 478（60.1） | 105（64.8） |  |
| pneumonia |  |  | 0.350 |
| No | 478（60.1） | 91（56.2） |  |
| Yes | 317（39.9） | 71（43.8） |  |
| Changes in the structure of the heart |  |  | 0.024 |
| No | 507（63.8） | 88（54.3） |  |
| Yes | 288（36.2） | 74（45.7） |  |
| Rhythm |  |  | 0.775 |
| Sinus | 578（72.7） | 116（71.6） |  |
| Non-sinus | 217（27.3） | 46（28.4） |  |
| Myocardial bridge |  |  | 0.751 |
| No | 774（97.4） | 157（96.9） |  |
| Yes | 21（2.6） | 5（3.1） |  |
| CTO |  |  | 0.174 |
| No | 776（97.6） | 154（95.1） |  |
| Yes | 19（2.4） | 8（4.9） |  |
| Gastrointestinal bleeding |  |  | 0.305 |
| No | 744（93.6） | 148（91.4） |  |
| Yes | 51（6.4） | 14（8.6） |  |
| heart failure |  |  | 0.284 |
| No | 670（84.3） | 131（80.9） |  |
| Yes | 125（15.7） | 31（19.1） |  |
| Grading of cardiac function |  |  | 0.023 |
| 1-2 | 655（82.4） | 121（74.7） |  |
| 3-4 | 140（17.6） | 41（25.3） |  |
| Diffuse coronary changes |  |  | 0.189 |
| No | 657（82.7） | 127（78.4） |  |
| Yes | 137（17.3） | 35（21.6） |  |
| Pathological Q waves |  |  | 0.234 |
| No | 695（87.4） | 136（84） |  |
| Yes | 100（12.6） | 26（16） |  |
| Number of diseased blood vessels |  |  | 0.101 |
| Less than 3 sticks | 365（45.9） | 63（38.9） |  |
| 3 or more | 430（54.1） | 99（61.1） |  |
| Criminal coronary vessels |  |  | 0.682 |
| Single | 601（75.6） | 120（74.1） |  |
| Multi-branch | 194（24.4） | 42（25.9） |  |
| Drinking |  |  | 0.559 |
| Yes | 314（39.5） | 60（37） |  |
| No | 481（60.5） | 102（63） |  |
| Smoking |  |  | 0.252 |
| Yes | 422（53.1） | 78（48.1） |  |
| No | 373（46.9） | 84（51.9） |  |
| Number of days in hospital |  |  | 0.008 |
| Less than 7 days | 204（25.7） | 58（35.8） |  |
| Greater than or equal to 7 days | 591（74.3） | 104（64.2） |  |
| Sleeping |  |  | 0.498 |
| normal | 337（42.4） | 64（39.5） |  |
| abnormal | 458（57.6） | 98（60.5） |  |
| TIMI blood flow |  |  | 0.175 |
| 2 | 10（1.3） | 5（3.1） |  |
| 3 | 785（98.7） | 157（96.9） |  |
| Number of stents | 1（1，2） | 1（1，2） | 0.184 |
| Rotary grinding |  |  | 0.657 |
| No | 754（94.8） | 155（95.7） |  |
| Yes | 41（5.2） | 7（4.3） |  |
| Intraoperative hypotension |  |  | 0.321 |
| No | 733（92.2） | 153（94.4） |  |
| Yes | 62（7.8） | 9（5.6） |  |
| Intraoperative arrhythmia |  |  | 0.634 |
| No | 735（92.5） | 148（91.4） |  |
| Yes | 60（7.5） | 14（8.6） |  |
| Intraoperative vascular ultrasound |  |  | 0.250 |
| No | 750（94.3） | 149（92） |  |
| Yes | 45（5.7） | 13（8） |  |
| red blood cells | 4.3398±0.65757 | 4.3255±0.63570 | 0.800 |
| Hematocrit | 0.404±0.061 | 0.3995±0.0657 | 0.398 |
| platelet | 181.708±68.231 | 180.667±71.101 | 0.860 |
| lymphocyte | 1.5282±0.6485 | 1.4383±0.67614 | 0.110 |
| monocyte | 0.5001±0.22689 | 0.5152±0.26041 | 0.450 |
| white blood cell | 7.8488±3.15126 | 7.6274±2.87708 | 0.409 |
| Neutrophils | 5.6278±3.06263 | 5.4995±2.71382 | 0.621 |
| Neutrophil ratio | 0.6953±0.10987 | 0.7022±0.10759 | 0.463 |
| haemoglobin | 127.8042±21.82393 | 122.9383±18.78439 | 0.008 |
| INR | 1.07±0.315 | 1.0942±0.30764 | 0.442 |
| PLR | 119.13（87.31，170.00） | 129.26（92.7425，183.465） | 0.155 |
| NLR | 3.29（2.31，5.11） | 3.49（2.36，6.15） | 0.332 |
| D-dimer | 0.82（0.59，1.31） | 0.88（0.59，1.36） | 0.535 |
| D-dimer peak | 1.00（0.65，1.56） | 0.98（0.6675，1.6075） | 0.885 |
| CRP | 3.90（1.60，9.69） | 5.87（2.14，19.52） | 0.002 |
| myoglobin | 50.97（32.28，92.39） | 52.51（34.835，82.525） | 0.808 |
| creatine kinase | 3.21（1.75，10.29） | 3.585（2.35，6.76） | 0.149 |
| Troponin T | 0.241（0.095，1.201） | 0.791（0.190，1.935） | <0.001 |
| BNP | 521.30（152.59，1313.6） | 649.835（226.4225，1238.125） | 0.106 |
| homocysteine | 16.0614±7.0468 | 16.4481±9.4179 | 0.550 |
| Fasting glucose | 8.013±3.417 | 9.102±4.924 | 0.008 |
| creatinine | 76.6（65，94.4） | 81.29（64.925，109.69） | 0.107 |
| Glomerular filtration rate | 83.88(67.43,96.15) | 76.97(57.11,96.52) | 0.037 |
| urea | 5.75（4.55，7.64） | 6.27（4.60，8.10） | 0.122 |
| Cystatin | 1.10（0.93，1.37） | 1.21(1.01，1.60) | <0.001 |
| uric acid | 348.3(291.60,426.42) | 357.99(294.40,427.53) | 0.725 |
| lactic acid | 1.71（1.25，2.4） | 1.95（1.49，2.59） | 0.005 |
| phosphorus | 0.95（0.86，1.08） | 0.95（0.86，1.07） | 0.416 |
| magnesium | 0.84（0.80，0.90） | 0.84（0.79，0.89） | 0.783 |
| potassium | 3.93（3.64，4.28） | 3.89（3.60，4.21） | 0.457 |
| sodium | 138.37（136.00，141.40） | 138.60(136.46,141.33) | 0.916 |
| calcium | 2.28(2.19,2.37) | 2.27(2.18,2.35) | 0.177 |
| Total bilirubin | 13.06（9.6，16.84） | 11.91（9.2，16.51） | 0.093 |
| Direct bilirubin | 3.2（2.3，4.52） | 2.92（2.57，3.9） | 0.637 |
| albumin | 38.5(35.70,41.1) | 37.50(35.20,40.48) | 0.041 |
| ALT | 17（10，27.7） | 16（10.8，25.23） | 0.937 |
| Lipoprotein A | 193.62（86.19，371.5） | 187.30(87.58，339.97） | 0.838 |
| TC | 4.31(3.45,5.24) | 4.73(3.78,5.32) | 0.012 |
| TG | 1.49(1.08,2.04) | 1.73(1.29,2.19) | <0.001 |
| Glycated hemoglobin | 7.30（6.59，8.13） | 7.625（6.49，9.125） | 0.036 |
| AST | 30.8（25，41） | 30.15（25.91，41.7） | 0.536 |
| HDL | 1.18(1.04,1.40) | 1.06(0.88,1.28) | <0.001 |
| LDL | 2.25(1.71,2.85) | 2.46(1.88,3.01) | 0.006 |
| heparin |  |  | 0.750 |
| No | 5（0.6） | 2（1.2） |  |
| Yes | 790（99.4） | 160（98.8） |  |
| Antiplatelet drug types |  |  | 0.383 |
| 1 | 6（0.8） | 3（1.9） |  |
| 2 | 789（99.2） | 159（98.1） |  |
| Statins |  |  | 1 |
| No | 4（0.5） | 1（0.6） |  |
| Yes | 791（99.5） | 161（99.4） |  |
| Proton pump inhibitors |  |  | 0.774 |
| No | 54（6.8） | 10（6.2） |  |
| Yes | 741（93.2） | 152（93.8） |  |
| ACEI/ARB/ARNI |  |  | 0.021 |
| No | 373（46.9） | 60（37.0） |  |
| Yes | 422（53.1） | 102（63） |  |
| B-blockers |  |  | 0.043 |
| No | 185（23.3） | 26（16） |  |
| Yes | 610（76.7） | 136（84） |  |
| CCB |  |  | 0.834 |
| No | 527（66.3） | 106（65.4） |  |
| Yes | 268（33.7） | 56（34.6） |  |
| Nitrates |  |  | 0.581 |
| No | 106（13.3） | 19（11.7） |  |
| Yes | 689（86.7） | 143（88.3） |  |
| Hypoglycemic agents |  |  | 0.057 |
| No | 579（72.8） | 106（65.4） |  |
| Yes | 216（27.2） | 56（34.6） |  |
