# Supplementary material for: Development and validation of a machine learning-based readmission risk prediction model for non-ST elevation myocardial infarction patients after percutaneous coronary intervention
Source: Sci Rep. 2024 Jun 11;14:13393. doi: 10.1038/s41598-024-64048-x (PMC11166920; doi:10.1038/s41598-024-64048-x)
Supplement: Supplementary file 9 — Supplementary Information 9. [file 41598_2024_64048_MOESM9_ESM.docx]

**S3 Baseline characteristics for all variables**

| Readmission of patients with NSTEMI after PCI surgery (validation) | | | |
| --- | --- | --- | --- |
| variable | non-readmission(N=342) | re-admission (N=64) | P |
| Sex（female） | 95（27.8） | 25（39.1） | 0.069 |
| Ethnicity (Han) | 342（100） | 63（98.4） | 0.158 |
| BMI | 23.67±3.00 | 23.31±3.04 | 0.374 |
| Age | 65.55±11.166 | 70.47±11.266 | 0.001 |
| Admission room |  |  | 0.710 |
| emergency | 43（12.6） | 7（10.9） |  |
| Outpatient | 297（86.8） | 56（87.5） |  |
| other | 2（0.6） | 1（1.6） |  |
| Discharge outcomes |  |  | <0.001 |
| Ease | 299（87.4） | 45（70.3） |  |
| Non-ease | 43（12.6） | 19（29.7） |  |
| medicare |  |  | 0.394 |
| No | 22（6.4） | 6（9.4） |  |
| Yes | 320（93.6） | 58（90.6） |  |
| Education |  |  | 0.026 |
| High school or less | 249（72.8） | 55（85.9） |  |
| High school and above | 93（27.2） | 9（14.1） |  |
| marriage |  |  | 0.535 |
| married | 325（95.0） | 59（92.2） |  |
| unmarried | 17（5.0） | 5（7.8） |  |
| Systolic blood pressure | 127(113.75，145) | 126.5(114.5，152.25) | 0.382 |
| Diastolic blood pressure | 77.5(68，86) | 77(69.25，82.75) | 0.500 |
| heart rate | 75(68.00，84.25） | 76.5(67.00，82.75) | 0.838 |
| body temperature | 36.50(36.50,36.60) | 36.50(36.50,36.60) | 0.703 |
| Number of breaths | 20.00(20.00,21.00) | 20.00(20.00,21.00) | 0.285 |
| mode |  |  | 0.001 |
| Walking | 304（88.9） | 47（73.4） |  |
| No-walking | 38（11.1） | 17（26.6） |  |
| awareness |  |  | 0.288 |
| awake | 334（97.7） | 61（95.3） |  |
| Non-awake | 8（2.3） | 3（4.7） |  |
| Communication skills |  |  | 0.015 |
| Good | 341（99.7） | 62（96.9） |  |
| Poor | 1（0.3） | 2（3.1） |  |
| Ejection fraction |  |  | 0.405， |
| ≥50% | 287（83.9） | 51（79.7） |  |
| <50% | 55（16.1） | 13（20.3） |  |
| diabetes |  |  | 0.048 |
| No | 216（63.2） | 32（50.0） |  |
| Yes | 126（36.8） | 32（50.0） |  |
| hypertension |  |  | 0.312 |
| No | 157（45.9） | 25（39.1） |  |
| Yes | 185（54.1） | 39（60.9） |  |
| Stroke |  |  | 0.008 |
| No | 271（79.2） | 41（64.1） |  |
| Yes | 71（20.8） | 23（35.9） |  |
| Peripheral vascular lesions |  |  | 0.480 |
| No | 139（40.6） | 23（35.9） |  |
| Yes | 203（59.4） | 41（64.1） |  |
| pneumonia |  |  | 0.736 |
| No | 216（63.2） | 39（60.9） |  |
| Yes | 126（36.8） | 25（39.1） |  |
| Changes in the structure of the heart |  |  | 0.027 |
| No | 226（66.1） | 33（55.6） |  |
| Yes | 116（33.9） | 31（44.4） |  |
| Rhythm |  |  | 0.830 |
| Sinus | 245（71.6） | 45（70.3） |  |
| Non-sinus | 97（28.4） | 19（29.7） |  |
| Myocardial bridge |  |  | 0.462 |
| No | 332（97.1） | 61（95.3） |  |
| Yes | 10（2.9） | 3（4.7） |  |
| CTO |  |  | 0.181 |
| No | 332（97.1） | 60（93.8） |  |
| Yes | 10（2.9） | 4（6.2） |  |
| Gastrointestinal bleeding |  |  | 0.508 |
| No | 318（93.0） | 58（90.6） |  |
| Yes | 24（7.0） | 6（9.4） |  |
| heart failure |  |  | 0.679 |
| No | 280（81.9） | 51（79.7） |  |
| Yes | 62（18.1） | 13（20.3） |  |
| Grading of cardiac function |  |  | 0.036 |
| 1-2 | 284（83.0） | 46（71.9） |  |
| 3-4 | 58（17.0） | 18（28.1） |  |
| Diffuse coronary changes |  |  | 0.634 |
| No | 276（80.7） | 50（78.1） |  |
| Yes | 66（19.3） | 14（21.9） |  |
| Pathological Q waves |  |  | 0.470 |
| No | 295（86.3） | 53（82.8） |  |
| Yes | 47（13.7） | 11（17.2） |  |
| Number of diseased blood vessels |  |  | 0.146 |
| Less than 3 sticks | 162（47.4） | 24（37.5） |  |
| 3 or more | 180（52.6） | 40（62.5） |  |
| Criminal coronary vessels |  |  | 0.726 |
| Single | 253（74.0） | 46（71.9） |  |
| Multi-branch | 89（26.0） | 18（28.1） |  |
| Drinking |  |  | 0.611 |
| Yes | 129（37.7） | 22（34.4） |  |
| No | 213（62.3） | 42（65.6） |  |
| Smoking |  |  | 0.398 |
| Yes | 180（52.6） | 30（46.9） |  |
| No | 162（47.4） | 34（53.1） |  |
| Number of days in hospital |  |  | 0.027 |
| Less than 7 days | 83（24.3） | 24（37.5） |  |
| Greater than or equal to 7 days | 259（75.7） | 40（62.5） |  |
| Sleeping |  |  | 0.632 |
| normal | 150（43.9） | 26（40.6） |  |
| abnormal | 192（56.1） | 38（59.4） |  |
| TIMI blood flow |  |  | 0.225 |
| 2 | 5（1.5） | 3（4.7） |  |
| 3 | 337（98.5） | 61（95.3） |  |
| Number of stents | 1.00(1.00,2.00) | 1.00(1.00,2.00) | 0.219 |
| Rotary grinding |  |  | 0.618 |
| No | 323（94.4） | 62（96.9） |  |
| Yes | 19（5.6） | 2（3.1） |  |
| Intraoperative hypotension |  |  | 0.571 |
| No | 316（92.4） | 61（95.3） |  |
| Yes | 26（7.6） | 3（4.7） |  |
| Intraoperative arrhythmia |  |  | 0.394 |
| No | 320（93.6） | 58（90.6） |  |
| Yes | 22（6.4） | 6（9.4） |  |
| Intraoperative vascular ultrasound |  |  | 0.200 |
| No | 324（94.7） | 58（90.6） |  |
| Yes | 18（5.3） | 6（9.4） |  |
| red blood cells | 4.37±0.70 | 4.33±0.85 | 0.726 |
| Hematocrit | 0.40±0.062 | 0.40±0.077 | 0.879 |
| platelet | 189.56±59.33 | 180.38±63.86 | 0.262 |
| lymphocyte | 1.54±0.62 | 1.47±0.56 | 0.387 |
| monocyte | 0.50±0.23 | 0.52±0.19 | 0.635 |
| white blood cell | 7.84±2.79 | 7.76±3.22 | 0.833 |
| Neutrophils | 5.64±2.74 | 5.54±3.13 | 0.794 |
| Neutrophil ratio | 0.70±0.11 | 0.68±0.11 | 0.304 |
| haemoglobin | 129.49±21.35 | 121.95±27.57 | 0.041 |
| INR | 1.08±0.19 | 1.09±0.12 | 0.598 |
| PLR | 127.08（92.32，174.26） | 121.28(95.91，158.35） | 0.605 |
| NLR | 3.27（2.28，5.53） | 3.14（2.26，5.44） | 0.898 |
| D-dimer | 0.85（0.64，1.19） | 0.88（0.66，1.12） | 0.945 |
| D-dimer peak | 0.91（0.68,1.40） | 1.00（0.69,1.66） | 0.351 |
| CRP | 4.16(2.03，8.08） | 6.53(2.71，8.91） | 0.019 |
| myoglobin | 52.45（33.61,97.00） | 57.90(33.66,126.70） | 0.495 |
| creatine kinase | 3.22（1.57，14.59） | 3.91（1.98，13.75） | 0.569 |
| Troponin T | 0.23（0.09，1.08） | 0.34（0.24，1.29） | <0.001 |
| BNP | 684.0(285.88,2016.25） | 573.9(261.00,2035.00） | 0.960 |
| homocysteine | 16.05±7.83 | 16.24±7.80 | 0.859 |
| Fasting glucose | 8.04±3.18 | 9.12±3.45 | 0.015 |
| creatinine | 77（65.75，94.03） | 84.3(61.02，97.45） | 0.700 |
| Glomerular filtration rate | 82.43(65.54,96.58) | 79.23(59.75,99.50) | 0.346 |
| urea | 5.90（4.63，7.50） | 6.39（4.87，8.34） | 0.147 |
| Cystatin | 1.10（0.96，1.34） | 1.215（1.03，1.61） | 0.011 |
| uric acid | 348.12(285.95,423.57) | 353.14(283.75,431.28) | 0.856 |
| lactic acid | 1.40（1.1，1.8） | 1.88（1.3125，2.59） | <0.001 |
| phosphorus | 0.96(0.86,1.09) | 0.96(0.86,1.08) | 0.891 |
| magnesium | 0.85(0.79,0.90) | 0.84(0.79,0.90) | 0.772 |
| potassium | 3.90(3.60,4.22) | 3.91(3.50,4.29) | 0.866 |
| sodium | 138.00(135.00,142.62) | 139.00(137.00,142.00) | 0.291 |
| calcium | 2.28(2.20,2.36) | 2.28(2.19,2.34) | 0.501 |
| Total bilirubin | 13.25（9.78，17.43） | 11.81(8.88，16.08） | 0.057 |
| Direct bilirubin | 3.36（2.15，4.87） | 3.0(2.04，3.97） | 0.192 |
| albumin | 38.45(36.10,41.33) | 37.25(34.00,40.73) | 0.036 |
| ALT | 18.40(10.23，35.7） | 17.95（12.05，28.61） | 0.989 |
| Lipoprotein A | 205.14（105.89，384.96） | 218.18(87.08，486.83) | 0.973 |
| TC | 4.25(3.67,5.02) | 4.68(3.81,5.52) | 0.040 |
| TG | 1.45(1.06,1.96) | 1.64(1.39,2.19) | 0.007 |
| Glycated hemoglobin | 6.99(6.38，7.95） | 7.58(6.99，8.51） | 0.023 |
| AST | 26(19.00，44.10） | 25.8(19.28，35.18） | 0.560 |
| HDL | 1.20(1.06,1.39) | 1.04(0.86,1.30) | <0.001 |
| LDL | 2.21(1.75,2.75) | 2.36(1.93,3.11) | 0.032 |
| heparin |  |  | 0.498 |
| No | 3（0.9） | 1（1.6） |  |
| Yes | 339（99.1） | 63（98.4） |  |
| Antiplatelet drug types |  |  | 0.403 |
| 1 | 2（0.6） | 1（1.6） |  |
| 2 | 789（99.4） | 159（98.4） |  |
| Statins |  |  | 1 |
| No | 2（0.6） | 0（0） |  |
| Yes | 791（99.4） | 161（100） |  |
| Proton pump inhibitors |  |  | 0.843 |
| No | 27（7.9） | 4（6.3） |  |
| Yes | 315（92.1） | 60（93.7） |  |
| ACEI/ARB/ARNI |  |  | 0.032 |
| No | 162（47.4） | 21（32.8） |  |
| Yes | 180（52.6） | 43（67.2） |  |
| B-blockers |  |  | 0.027 |
| No | 99（28.9） | 10（15.6） |  |
| Yes | 243（71.1） | 54（84.4） |  |
| CCB |  |  | 0.652 |
| No | 229（67） | 41（64.1） |  |
| Yes | 113（33） | 23（35.9） |  |
| Nitrates |  |  | 0.371 |
| No | 46（13.5） | 6（9.4） |  |
| Yes | 296（86.5） | 58（90.6） |  |
| Hypoglycemic agents |  |  | 0.141 |
| No | 250（73.1） | 41（64.1） |  |
| Yes | 92（26.9） | 23（35.9） |  |
