# Supplementary material for: Development and validation of a machine learning-based readmission risk prediction model for non-ST elevation myocardial infarction patients after percutaneous coronary intervention
Source: Sci Rep. 2024 Jun 11;14:13393. doi: 10.1038/s41598-024-64048-x (PMC11166920; doi:10.1038/s41598-024-64048-x)
Supplement: Supplementary file 10 — Supplementary Information 10. [file 41598_2024_64048_MOESM10_ESM.docx]

**S4 Standard or statistical parameters of three variable screening methods**

| Variable selection method | Standard or statistical parameter |
| --- | --- |
| Univariate and multifactorial logistic regression | *P*<0.05 |
| Lasso regression | 𝜆= 0.01114426 |
| Random forest | N=500 |
