# Supplementary material for: Development and validation of a machine learning-based readmission risk prediction model for non-ST elevation myocardial infarction patients after percutaneous coronary intervention
Source: Sci Rep. 2024 Jun 11;14:13393. doi: 10.1038/s41598-024-64048-x (PMC11166920; doi:10.1038/s41598-024-64048-x)
Supplement: Supplementary file 11 — Supplementary Information 11. [file 41598_2024_64048_MOESM11_ESM.docx]

**S5 The standard or statistical parameters of the machine learning method that builds the model respectively**

| Prediction model | Standard or statistical parameter |
| --- | --- |
| LR | p<0.05 |
| DT | minsplit=20,cp=0.01 |
| RF | ntree=500,mtry=3 |
| SVM | cost=1,kernel="linear" |
| XGBoost | max_depth=3,eta=0.3,nrounds=100 |
| AdaBoost | mfinal = 100, coeflearn = 'Breiman' |
